# Identification of Body Color-Related QTLs in Leopard Coral Grouper (*Plectropomus leopardus*) Based on GWAS and the HSV Color System

**DOI:** 10.3390/ani16111627

**Published:** 2026-05-27

**Authors:** Yang Yang, Tong Wang, Leilei Zeng, Fuli Luo, Zhennian Chen, Jingfang Chen, Zining Meng, Xiaochun Liu

**Affiliations:** 1 College of Fisheries and Life Science, Hainan Tropical Ocean University, Sanya 572000, China; yangy0103@foxmail.com; 2 Key Laboratory of Tropical Marine Fish Germplasm Innovation and Utilization, Ministry of Agriculture and Rural Affairs, Hainan Engineering Research Center for Germplasm Innovation and Utilization, Hainan Chenhai Aquatic Co., Ltd., Sanya 572025, China; luofuli66@126.com (F.L.); chenzhennian0111@126.com (Z.C.); 3 State Key Laboratory of Biocontrol/Southern Marine Science and Engineering Guangdong Laboratory (Zhuhai)/Guangdong Provincial Key Laboratory of Aquatic Economic Animals, School of Life Sciences, Sun Yat-Sen University, Guangzhou 510275, China; sysuwangtong@163.com (T.W.); zlei5716@163.com (L.Z.); mengzn@mail.sysu.edu.cn (Z.M.); lsslxc@mail.sysu.edu.cn (X.L.); 4 Yazhou Bay Innovation Institute, Sanya 572025, China

**Keywords:** *Plectropomus leopardus*, body color, GWAS

## Abstract

Leopard coral grouper (*Plectropomus leopardus*), a member of the *Epinephelidae* family, is characterized by its distinct red coloration and excellent meat quality, making it one of the most premium species in the grouper market. Body color is an important economic trait for leopard coral grouper because it is an important factor in determining market price. In order to improve the traits in leopard coral grouper, the regulatory mechanism of body color and pigmentation is essential to explore. In the research, QTLs associated with body color in leopard coral grouper were detected using genome-wide association study analysis. A mixed population derived from 12 female and 12 male wild individuals with significant color differences was established. Meanwhile, the HSV (Hue, Saturation, Value) color model was employed to quantify leopard coral grouper body color as continuous variables. In the results, a total of 18 SNPs associated with the body color of the leopard coral grouper were discovered. Through functional annotation, we identified four candidate genes associated with body color: ASAP2, NLRC3, ALDH18A1, and E2F4. These genes were involved in chromatophore distribution, contraction and dilation, carotenoid oxidation, pigment cell proliferation and development, and immune-related processes. These findings uncovered new genetic loci and regulatory mechanisms for body color, providing a genetic basis for understanding pigmentation regulation and supporting marker-assisted selective breeding in leopard coral grouper.

## Simple Summary

Body color is an important economic trait for leopard coral grouper, as it directly affects market price. In order to improve the traits in leopard coral grouper, the regulatory mechanism of body color is essential to explore. In the research, the QTLs associated with body color in leopard coral grouper were detected using genome-wide association study analysis with a mixed population. Meanwhile, the HSV (Hue, Saturation, Value) color model was employed to quantify leopard coral grouper body color as continuous variables. In the results, four candidate genes associated with body color were found; these genes were involved in chromatophore distribution, contraction and dilation, carotenoid oxidation, pigment cell proliferation and development, and immune-related processes.

## 1. Introduction

Groupers (*Epinephelidae*, *Perciformes*) inhabit coral reef ecosystems, which comprise more than 160 species in 16 genera [1]. They are also commercially important fishery and aquaculture species worldwide. Groupers are extensively farmed, with approximately 47 species currently cultivated [2]. Market demand for grouper is increasing sharply all over the world. In China, the annual output of groupers has reached ~260,000 tons in 2024, as reported in the China Fishery Statistical Yearbook. The leopard coral grouper (*Plectropomus leopardus*) is the oldest species in the grouper family [3] and is mainly distributed in the Western Pacific regions from Western Australia eastward to the Caroline Islands and Fiji and from southern Japan to Queensland, Australia. The wild populations of this fish have decreased due to the destruction of spawning aggregations and overfishing; protection measures for this fish have been strongly appealed to set up in IUCN https://www.iucnredlist.org/species/44684/100462709 (accessed on 12 May 2025). Like other groupers, leopard coral grouper is a protogynous hermaphrodite, meaning fish typically start as a female and later change to male [4]. The leopard coral grouper has been widely cultured because of its high nutritional value, tender flesh, and beautiful body color. Body color is an important economic trait for leopard coral grouper. The colors of the species are various, including red, grey, black, pink, and white. Body color determines the commercial quality of leopard coral grouper, because the Chinese market favors a bright red body color. The bright red fish is sold at twice the price of black fish in Chinese markets.

Body color is a trait controlled by numerous genes. Typical genes associated with physiological color were melanin-concentrating hormone (MCH), α-melanocyte-stimulating hormone (α-MSH), agouti-signaling protein (ASP), MCH receptor, and melanocortin receptors (MCRs) [5]. In previous studies, some genes associated with differences in body color were predicted in fish. For example, SLC45A2 and MITF were detected as body color-related genes in Malaysian red tilapia (*Oreochromis* spp.) [6], and genes associated with pigment synthesis, carotenoid metabolism, and chromatophore proliferation and apoptosis were detected in crucian carp with different colors [7]. In leopard coral groupers, the content and proportion of carotenoids are important factors for body color. The different proportions of carotenoids such as tunaxanthin, astaxanthin, and tunaxanthin determine the body color of this species. In redfish, the astaxanthin contents reach 81.2%, which is higher than in gray and brown coral by 100-fold and 4-fold, respectively [8]. A comparative transcriptome analysis detected 38 candidate genes underlying the mechanism of color and pigmentation [3]. A total of 74 single nucleotide polymorphisms and one indel were detected in the complete mitochondrial genome of red and grey fish [9]. In order to improve the traits in leopard coral grouper, the molecular mechanism of regulation of body color is essential to explore. Recent research showed that scavenger receptor genes play important roles in carotenoid pigment deposition, which led to the change of skin brightness and carotenoid levels in leopard coral grouper [10]. A genome-wide association study analysis of the black color trait in the leopard coral grouper discovered multiple candidate genes associated with multiple signaling pathways related to melanin synthesis [11]. ATAC-seq and RNA-seq analyses revealed epigenetic regulation of body color variation in the leopard coral grouper associated with melanogenesis, the MAPK signaling pathway, lipid metabolism pathways, and immune-related signaling pathways [12].

Previous studies have mainly focused on the obvious color differences. In studies on body color in the leopard coral grouper, the binary classification method was commonly employed to quantify body color. In one study, researchers selected red and brown leopard coral groupers for transcriptomic analysis and identified genes related to carotenoid metabolism [13]. Recent research revealed that black pigmentation in leopard coral groupers was collectively regulated by multiple key genes based on binomial distribution [11]. In another study, researchers performed transcriptomic and metabolomic analyses on red and black leopard coral groupers, discovering genes associated with carotenoid uptake, transport, and accumulation, as well as arachidonic acid enrichment and melanin synthesis [14]. However, the degree of red is the major problem in leopard coral grouper, and there has been no effective approach to quantify it. In the research, the HSV (Hue, Saturation, Value) color system was adopted as a quantitative method to convert body color into variables. The HSV color system is more consistent with how humans describe and interpret colors. Recent research reported that eye color, quantified as continuous variables based on the HSV color system, was used in whole genome association studies, and three new loci related to eye color were discovered [15].

In this study we aimed to uncover the genetic architecture of color in leopard coral grouper using the HSV color system. This research will provide a genetic basis for the understanding of the regulative mechanism and genomic selection of the body color of the leopard coral grouper.

## 2. Materials and Methods

### 2.1. Experimental Animals

The parental fish, consisting of 12 females and 12 males, were obtained from Chenhai Aquatic Co., Ltd. (Sanya, China). 400 g of eggs from all females and 400 μL of sperm from all males were uniformly mixed in a bucket, added to 15 L of seawater, and left to stand for 5 min. Subsequently, the semen was removed using clean water, artificial fertilization completed. The fertilized eggs were incubated in hatching apparatuses. The resultant offspring were then reared in indoor concrete ponds with dimensions of 14 m × 14 m × 2 m (length × width × depth) with low light intensity and managed following standard fishery culture protocols. All animal handling procedures were approved by the Institutional Animal Care and Use Committee of Hainan Tropical Ocean University.

### 2.2. Sampling and Photographic Data

Approximately eight months later, 307 individuals (243.4 ± 29.0 g) of F1 were randomly collected for this study. The photograph of the 307 offspring was recorded. The method of photographing was as follows: the fish were anesthetized with 100 ppm eugenol for 15–20 min before sampling to ensure that the pigment cells were fully expanded as much as possible. The fish was photographed as follows: photographs were taken under fixed conditions, including light source, illumination intensity, aperture, ISO, exposure time, and focal length, with a color temperature of 6000 K using DSLR Camera K50 (Pentax, Tokyo, Japan). Meanwhile, the right-sized caudal or dorsal fin of all individuals was collected and kept in 75% ethyl alcohol. Finally, those samples were removed from ethyl alcohol and transported to the laboratory −80 °C refrigerator until used for genomic DNA extraction.

### 2.3. DNA Extraction and Library Construction

**Figure 1:**
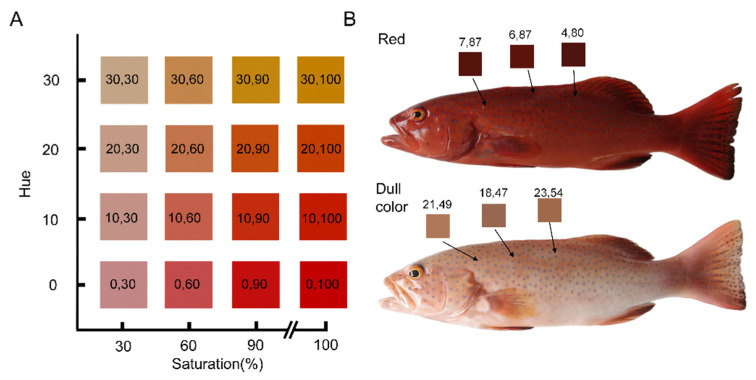
Schematic diagram of the HSV color system and sampling procedure for the leopard coral grouper. (**A**) shows a schematic diagram showing visual colors under different hue and saturation conditions; (**B**) illustrates the hue and saturation of two groupers with distinct body color and the sampling methodology for body color.

Genomic DNA of each sample was extracted using TIANamp Marine Animals DNA Kit (Tiangen Biotech, Beijing, China) following the manufacturer’s instructions. The quality and quantity of each DNA sample were assessed using Qubit 3.0 (Thermo Scientific, Waltham, MA, USA) and electrophoresis on 1% agarose gel. The HSV color system was used to quantify visual coloration as a continuous variable (Figure 1A). For the hue and saturation, the individuals were sampled using Just Color Picker v5.4.0 software https://annystudio.com/software/colorpicker/#download (accessed on 2 May 2025), and 3-5 points were randomly selected for measurement (Figure 1B). The hue and saturation values of each point were measured, and their mean values were defined as the hue and saturation of the individual. During sampling, pigmented spots on the body were excluded from color measurement.

### 2.4. Library Construction and Sequencing

DNA of all individuals was used for the preparation of ddRAD-seq libraries. Sequencing libraries were prepared using the two-enzyme modification of the original GBS protocol [16,17] and submitted to the Novogene Corporation (Beijing, China) for Illumina HiSeq PE150 (paired-end 150 bp) sequencing. Briefly, the procedures for ddRAD library construction were as follows: genomic DNA (gDNA) was digested in 96-well plates using the restriction enzymes *EcoRI* and *HaeIII*; the double-digested DNA fragments were then ligated with 25 pmol of A1 and A2 adapters per well to attach adapters to both ends of the DNA fragments. The DNA fragments were size-selected (400–600 bp) on a 1% agarose gel and purified using a PCR purification kit (NEB, Ipswich, MA, USA). Finally, the products were enriched and purified via PCR amplification, and fragment sizes were evaluated using a Bioanalyzer 2100 (Agilent, Santa Clara, CA, USA). The pooled libraries were adjusted to 3 nmol and sequenced on one lane of an Illumina HiSeq platform (Illumina, San Diego, CA, USA) with the PE-150.

### 2.5. SNP Discovery and Genotyping

Raw sequencing reads generated from genomic DNA were filtered and trimmed to obtain high-quality clean reads based on three stringent criteria. First, reads associated with barcode and adapter sequences were removed. Second, reads containing ≥10% unidentified nucleotides (N) were discarded. Third, low-quality reads with more than 50% of their bases exhibiting a Phred quality score of ≤20 were eliminated. In order to gain genetic variants, clean reads of each sample were aligned to the reference genome of leopard coral grouper [13] using the Bowtie2 v2.4.2 program with default parameters [18]. SNPs were called with minimum quality > 30 using BCFtools v1.8 programs: mpileup, call, and filter [19]. SNPs with depth < 3, minor allele frequency (MAF) < 0.05, missing position < 10%, and number of alleles > 2 were removed using VCFtools v0.1.17 [20].

### 2.6. GWAS Analysis of the Body Color Traits

The fish phenotypes from 307 individuals were analyzed using a mixed linear model (MLM) implemented in TASSEL5.0 [21]. Genotypic PCA data and population kinship data, both calculated using TASSEL 5.0, were used as covariates in the genome-wide association study. The genome-wide significance threshold was determined according to log10 (*p*-value) > 5 [22]. The QQ plots and Manhattan plots were all drawn with CMplot v4.5.1 software. The phenotypic variance explained (PVE) and *F* value were calculated using TASSEL5.0 with defaults.

### 2.7. Annotation

Functional annotation was performed on the significant SNP loci identified by GWAS analysis. The genomic positions of these SNPs were mapped against the genome annotation file (GFF), and the significant loci were categorized into intergenic regions, exonic regions, and intronic regions. For SNPs located in intergenic regions, their upstream and downstream genes were extracted as potential candidate genes. For SNPs located in exonic regions, their mutation types were determined and classified.

## 3. Results

### 3.1. Conversion of Visual Colors

**Figure 2:**
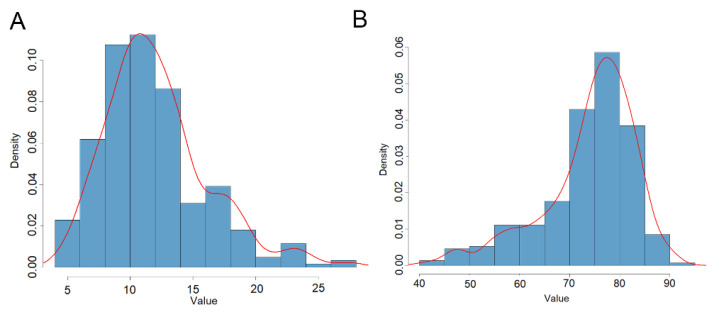
Histograms and kernel density estimate line of body color hue (**A**) and saturation (**B**) in the sampled population of leopard coral grouper.

The visual images were converted into data variables, and histograms were plotted. The results show that hue and saturation have been transformed into continuous variables and are approximately normally distributed (Figure 2). However, since brightness data is not directly related to color, this study uses hue and saturation for subsequent analysis. Figure 1B shows that red skin has lower hue values and higher saturation, whereas lighter skin has higher hue values and lower saturation.

### 3.2. Sequencing Data and Characterization of SNPs

After removing low-quality reads and SNP calling, a total of 109,338 genetic variants were obtained. In total, 89,037 SNPs and 20,301 indels were identified. Subsequent analyses will focus on the SNPs as the primary genetic markers. Among the identified SNPs, there were 61,879 transitions (TS) and 27,204 transversions (TV), yielding a TS/TV ratio of 2.27. Chromosome 2 contains the highest number of SNP loci, at 6349, while chromosome 24 contains the lowest, at 2965.

### 3.3. Analysis of Population Structure

**Figure 3:**
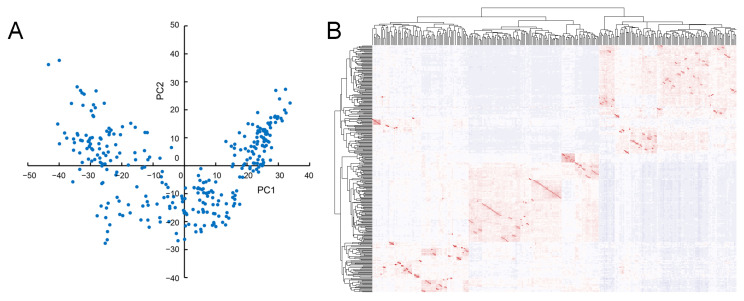
Population structure of the sampled leopard coral grouper. (**A**) shows PCA plot of the population used for the genome-wide association study; (**B**) shows kinship matrix plot of the population, where red indicates close genetic relatedness, while purple represents distant genetic relatedness.

Kinship and principal component analyses (PCA) were conducted to investigate the genetic relationships within the population and to utilize this genetic information as covariates in the genome-wide association study. The PCA results revealed no significant population stratification (Figure 3A). Furthermore, the majority of kinship values ranged from 0 to 0.5, indicating weak genetic relatedness among individuals (Figure 3B). Consequently, this constitutes an ideal cohort for GWAS. Subsequently, the top five PCs and kinship data were incorporated as covariates in the GWAS analyses.

### 3.4. Genome-Wide Association Analysis of Body Color

**Figure 4:**
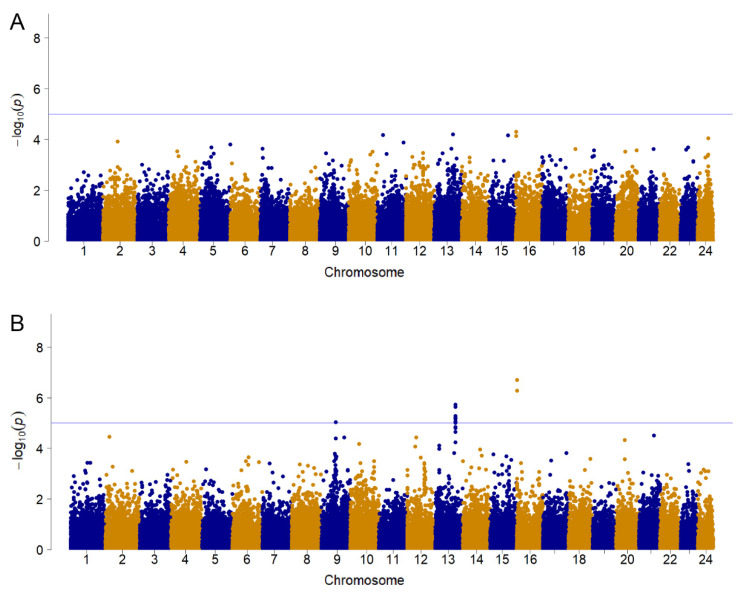
Manhattan plot of the genome-wide association study for body color in the leopard coral grouper. (**A**) indicates hue; (**B**) indicates saturation. The blue line represents the threshold line of significance.

Genome-wide association studies (GWAS) were conducted separately with Hue and Saturation as target traits. No SNPs were found to be significantly associated with Hue (Figure 4A). A total of 18 SNPs were identified to be significantly associated with Saturation. The strongest association signals were identified on chromosome 16 (Figure 4). Specifically, a tight cluster of SNPs (including S16_797851, S16_797853, and S16_797854) exhibited the highest statistical significance (log10 (*p*-value) = 6.71) and explained the largest proportion of the phenotypic variance (PVE = 9.19%). Furthermore, a secondary highly significant locus was detected on chromosome 13 (spanning approximately 24.32 Mb), containing multiple associated SNPs with PVE values ranging from 6.55% to 8.93%. An additional significant locus was also mapped to chromosome 9 (S09_17302939, PVE = 6.60%) (Table 1).

### 3.5. Identification of Candidate Genes Associated with Body Color

Functional annotation of the identified SNPs provided further insights into their genomic contexts and potential biological impacts. The highly significant variants on chromosome 16 map to an intergenic region flanked by the ASAP2 and NLRC3 genes. In contrast, the cluster of associated SNPs on chromosome 13 is predominantly located within exonic regions. While the majority of these exonic variants are synonymous mutations, one notable SNP (S13_24322603) results in a missense mutation within the ALDH18A1 gene; specifically, a glutamic acid is transversed to aspartic acid. Additionally, the significant variant on chromosome 9 (S09_17302939) is localized to an intronic region of the E2F4 gene (Table 2).

## 4. Discussion

Here, we performed a genome-wide mining for genes related to body coloration in *Plectropomus leopardus*. Given the traditional difficulties in reliably translating visual color traits into quantitative data, we employed the HSV color system to convert color profiles into continuous variables. This approach made GWAS for body color practically feasible, allowing us to successfully pinpoint key genetic loci and candidate genes responsible for color variation.

### 4.1. Quantitative Methods for Body Color Phenotype

Converting visual colors into a Bernoulli distribution is a commonly used method. Two relatively extreme color types are selected and divided into two groups, and colors are quantified as 0 and 1 to facilitate research on body color. For instance, a previous genome-wide association study on the storage root flesh color of sweet potato quantified the phenotype by simply categorizing the color into binary traits, purple and non-purple [23]. Another research study discovered a QTL associated with body color of pigs using GWAS analysis based on binary traits completely black and black with white patterns [24]. Alternatively, some research has classified color phenotypes into multiple ordinal categories or discrete ranks. A large-scale GWAS involving almost 195,000 individuals identified 50 previously unidentified genetic loci by categorizing eye color into one of seven distinct groups, ranging from blue to dark brown, based on visual color matching [25]. In a pericarp color analysis of rice (*Oryza sativa* L.), scoring systems were used for GWAS. The rice panel was visually sorted into eight pericarp groups (1 to 8 scoring system) having seeds with green, white, off-white, light red, red, dark red, brown, and black pericarp, respectively, and later into two groups (0 and 1 scoring system) having seeds with colored and white pericarp, respectively [26]. However, forcing color into a binomial distribution is equivalent to artificially reducing a complex quantitative trait to a qualitative one, thereby eradicating all intra-group phenotypic variation. Furthermore, ordinal categorization inherently assumes equidistant biological intervals between ranks, an assumption that rarely holds true in reality. Additionally, assigning color values through visual inspection is highly prone to bias, as human perception is significantly influenced by ambient light, background contrast, and inter-observer variability. In contrast, the HSV color space provides continuous quantitative data and employs a strictly objective valuation system. Such continuous variables are intrinsically more compatible with the linear mixed models commonly utilized in GWAS. This compatibility substantially enhances the sensitivity of association signals, enabling the detection of minor-effect polygenes even within relatively small sample sizes. Recent research reported the color of human eyes, quantified as a continuous variate based on the HSV color system, was used in a whole genome association study analysis, and three new loci related to eye color were discovered [15]. Thus, in the research, we used the HSV system to qualify the body color of leopard coral grouper.

### 4.2. Candidate Genes Associated with Body Color

Through functional annotation, we identified four candidate genes associated with body color: ASAP2, NLRC3, ALDH18A1, and E2F4. ASAP2 is a GTPase-activating protein (GAP) that controls cell morphology, cell migration, and spatial cellular distribution by regulating actin and cytoskeletal proteins [27]. In the regulation of body color in the leopard coral grouper, ASAP2 may play a regulatory role in the dispersion, migration, and spatial distribution of chromatophores. NLRC3 (NLR family CARD domain containing 3) was traditionally recognized as an intracellular negative regulator of innate immunity, functioning to prevent immune system overactivation, avoid autoimmune tissue damage, and maintain cellular homeostasis. However, recent studies have revealed a close potential relationship between NLRC3 and body coloration. In cichlid fish, a transcriptomic comparison of yellow and red skin regions demonstrated significant differential expression of NLRC3 between these distinct color zones, leading to the hypothesis that it may be involved in the metabolism and maintenance of carotenoid-dependent chromatophores [28]. Similarly, a study investigating the body color of rainbow trout (*Oncorhynchus mykiss*) compared the whole-skin transcriptomes of normal-colored and yellow mutant individuals. The results revealed highly significant differential expression of nlrc3-related genes between the skin tissues of the two phenotypes, suggesting a strong association with color variation [29]. ALDH18A1 (Aldehyde Dehydrogenase 18 Family Member A1) mediates the biosynthesis of arginine, which serves as the exclusive substrate for nitric oxide (NO) production [30,31]. In fish, NO acts as a critical signaling molecule that orchestrates the dispersion and aggregation of pigment granules within chromatophores [32]. Specifically, NO donors can sustain the dispersion of pigments such as melanin, whereas the inhibition of NO synthesis triggers pigment aggregation [33]. Additionally, ALDH18A1 functions as the rate-limiting enzyme in the de novo synthesis of proline. As a robust intracellular antioxidant, proline may buffer oxidative stress, thereby protecting carotenoids from oxidative depletion [34]. Consequently, the cellular proline level influences the concentration and composition of carotenoids, ultimately driving the phenotypic divergence of body coloration. E2F4 is a multifunctional transcription factor and mediates cell cycle arrest, prompting melanocytes to exit the proliferation phase and undergo functional differentiation, which is closely associated with the synthesis and accumulation of melanin [35]. Multiple studies have demonstrated that E2F4 and its homologous genes are closely associated with the proliferation of melanoma cells [36,37]. E2F4 may affect the number of melanocytes, thereby leading to body color variation in leopard coral grouper.

## 5. Conclusions

In the research, we adopted the HSV (Hue, Saturation, Value) color system to quantify the body color of leopard coral grouper as continuous variables. The color description provided by HSV is natural and highly intuitive for human observers. This method demonstrated excellent quantitative results, retained subtle color variation, and greatly improved the power of GWAS for detecting minor effect loci, solving the problem of inaccurate color quantification. For GWAS results, a total of 18 SNPs associated with the body color of leopard coral grouper were discovered. Through functional annotation, we identified four candidate genes associated with body color: ASAP2, NLRC3, ALDH18A1, and E2F4. These genes are involved in chromatophore distribution, regulation of pigment dispersion and aggregation, carotenoid oxidation, pigment cell proliferation and development, and immune-related processes. These findings uncovered new genetic loci and regulatory mechanisms for body color, providing a genetic basis for understanding pigmentation regulation and supporting marker-assisted selective breeding in leopard coral grouper.

## Figures and Tables

**Table 1:** Statistical information of SNPs associated with body color in the leopard coral grouper.

Traits	SNP	Chr	Location	F	Log10 (*p*-Value)	PVE (%)
Saturation	S16_797851	16	797,851	28.37	6.71	9.19
Saturation	S16_797853	16	797,853	28.37	6.71	9.19
Saturation	S16_797854	16	797,854	28.37	6.71	9.19
Saturation	S16_797856	16	797,856	26.28	6.28	8.52
Saturation	S13_24322516	13	24,322,516	13.78	5.72	8.93
Saturation	S13_24322507	13	24,322,507	23.20	5.63	7.52
Saturation	S13_24322510	13	24,322,510	23.20	5.63	7.52
Saturation	S13_24322522	13	24,322,522	21.50	5.28	6.97
Saturation	S13_24322468	13	24,322,468	21.22	5.22	6.88
Saturation	S13_24322582	13	24,322,582	20.96	5.16	6.79
Saturation	S13_24322603	13	24,322,603	20.96	5.16	6.79
Saturation	S13_24322532	13	24,322,532	20.49	5.06	6.64
Saturation	S09_17302939	9	17,302,939	20.35	5.03	6.60
Saturation	S13_24322528	13	24,322,528	20.20	5.00	6.55
Saturation	S13_24322565	13	24,322,565	20.20	5.00	6.55
Saturation	S13_24322570	13	24,322,570	20.20	5.00	6.55
Saturation	S13_24322579	13	24,322,579	20.20	5.00	6.55
Saturation	S13_24322594	13	24,322,594	20.20	5.00	6.55

SNP: Single nucleotide polymorphism; Chr: Chromosome; F: Degree of freedom; PVE: Phenotypic variance explained, corresponding to the marker R^2^ value from MLM.

**Table 2:** Functional annotation of genes associated with body coloration in the leopard coral grouper.

SNP	Reference Base	Mutated Base	Location	Codon Alteration	Amino Acid Statistics	Annotation
S16_797851	A	G	Intergenic region	/	/	Up: ASAP2 Down: NLRC3
S16_797853	G	A	Intergenic region	/	/	
S16_797854	A	T	Intergenic region	/	/	
S16_797856	G	A	Intergenic region	/	/	
S13_24322516	A	T	Exonic	GCA-GCT	A-A	ALDH18A1
S13_24322507	C	A	Exonic	GGC-GGA	G-G	
S13_24322510	T	A	Exonic	GCT-GCA	A-A	
S13_24322522	G	C	Exonic	GGG-GGC	G-G	
S13_24322468	C	G	Exonic	CTC-CTG	L-L	
S13_24322582	T	A	Exonic	TCT-TCA	S-S	
S13_24322603	A	C	Exonic	GAA-GAC	E-D	
S13_24322532	C	T	Exonic	CTG-TTG	L-L	
S13_24322528	G	A	Exonic	CAG-CAA	Q-Q	
S13_24322565	A	C	Exonic	AGA-CGA	R-R	
S13_24322570	A	G	Exonic	CAA-CAG	Q-Q	
S13_24322579	T	C	Exonic	CAT-CAC	H-H	
S13_24322594	A	G	Exonic	CAA-CAG	Q-Q	
S09_17302939	G	A	Intronic	/	/	E2F4

## Data Availability

All of raw sequencing data applied to GWAS deposited in genome sequence archive (GSA, Beijing, China). The accession numbers of raw data were SRX18501151_SRX18501457.
